# Supplementary material for: choros: correction of sequence-based biases for accurate quantification of ribosome profiling data
Source: bioRxiv. 2023 Feb 22:2023.02.21.529452. Preprint. [Version 1] doi: 10.1101/2023.02.21.529452 (PMC9980091; doi:10.1101/2023.02.21.529452)
Supplement: Supplement 1 [file NIHPP2023.02.21.529452v1-supplement-1.pdf]

bioRxiv preprint doi: https://doi.org/10.1101/2023.02.21.529452; this version posted February 22, 2023. The copyright holder for this preprint (which was not certified by peer review) is the author/funder, who has granted bioRxiv a license to display the preprint in perpetuity. It is made available under aCC-BY 4.0 International license.

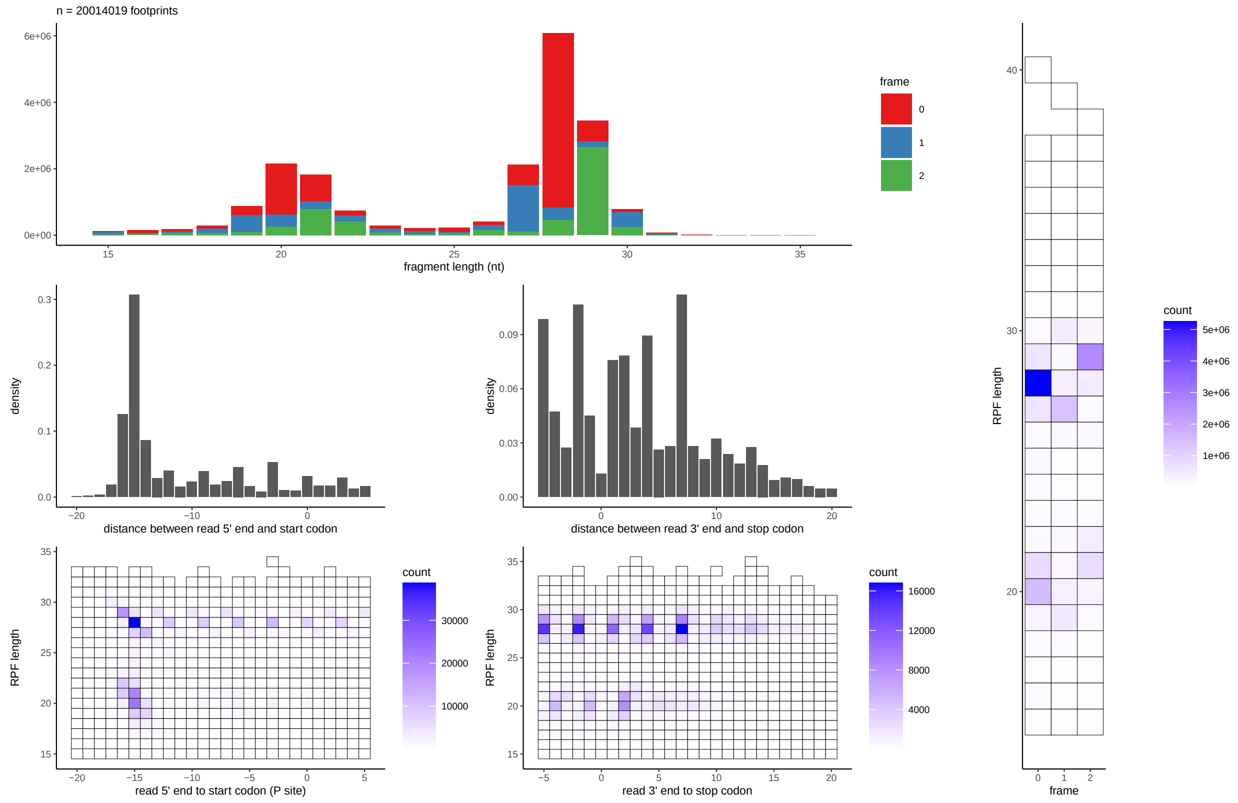

Supplementary Figure 1. Establishing A-site offset rules from a start codon metagene plot. This plot reflects data generated by Tunney et al. (2018) [13].

bioRxiv preprint doi: https://doi.org/10.1101/2023.02.21.529452; this version posted February 22, 2023. The copyright holder for this preprint (which was not certified by peer review) is the author/funder, who has granted bioRxiv a license to display the preprint in perpetuity. It is made available under aCC-BY 4.0 International license.

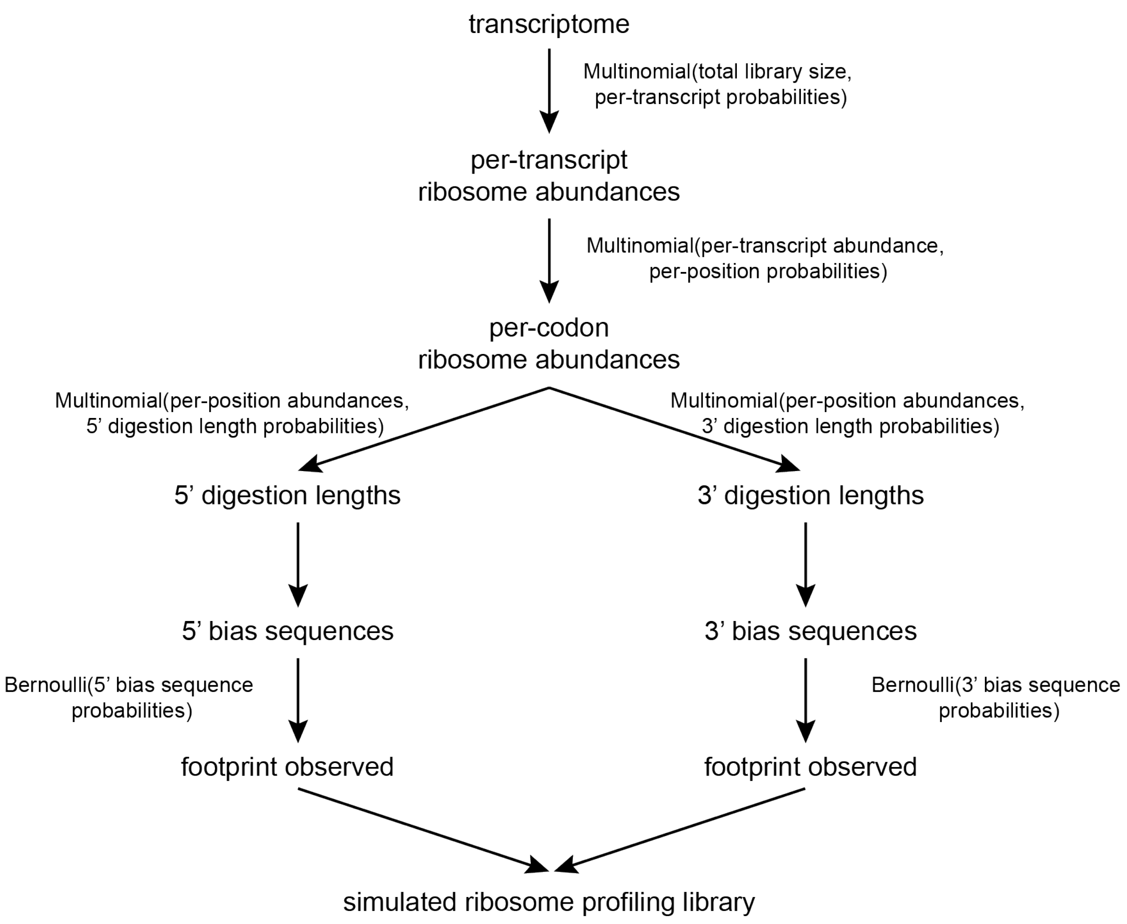

Supplementary Figure 2. Schematic diagram for the generation of simulated ribosome profiling datasets using `simRiboSeq`.

bioRxiv preprint doi: https://doi.org/10.1101/2023.02.21.529452; this version posted February 22, 2023. The copyright holder for this preprint (which was not certified by peer review) is the author/funder, who has granted bioRxiv a license to display the preprint in perpetuity. It is made available under aCC-BY 4.0 International license.

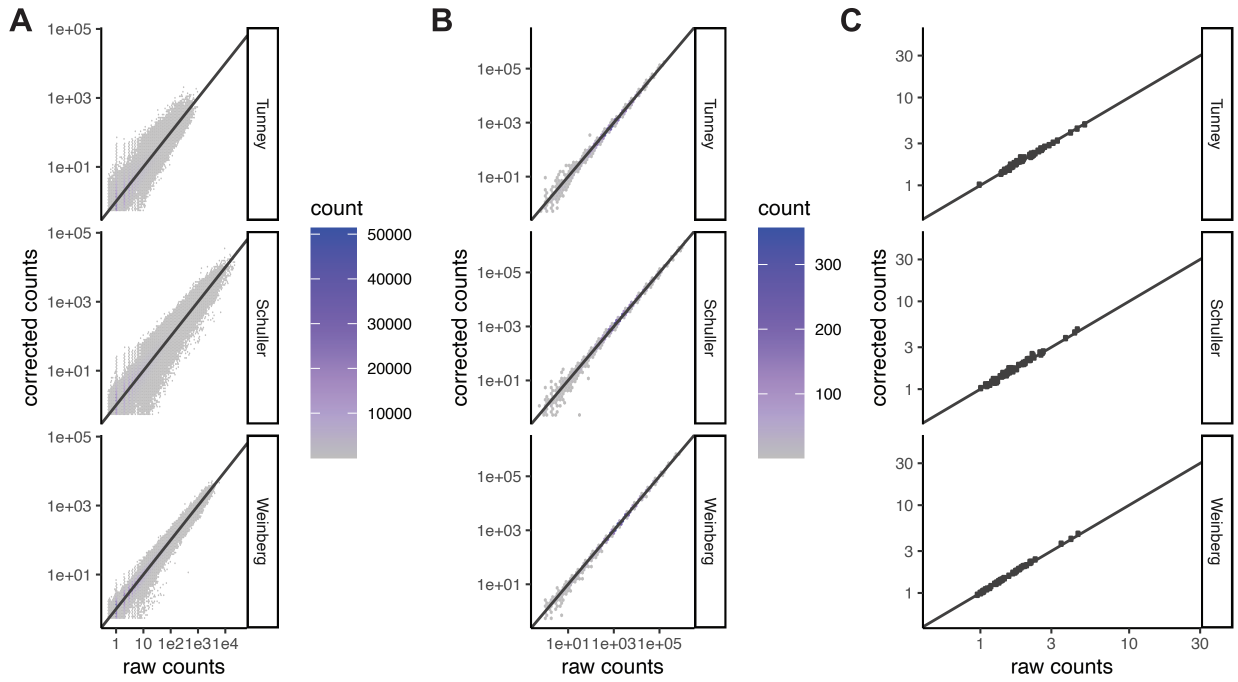

Supplementary Figure 3. Impact of bias correction on ribosome abundances. **(A)** Per-codon footprint counts, before and after bias correction. Footprint counts were summed by codon position. **(B)** Per-transcript footprint counts, before and after bias correction. **(C)** Codon pause scores, before and after bias correction. Codon pause scores were calculated as follows: footprint counts were aggregated by codon position and normalized to the mean read coverage by transcript (omitting the first and last 20 codons). Normalized footprint counts were averaged by codon identity across the transcriptome.

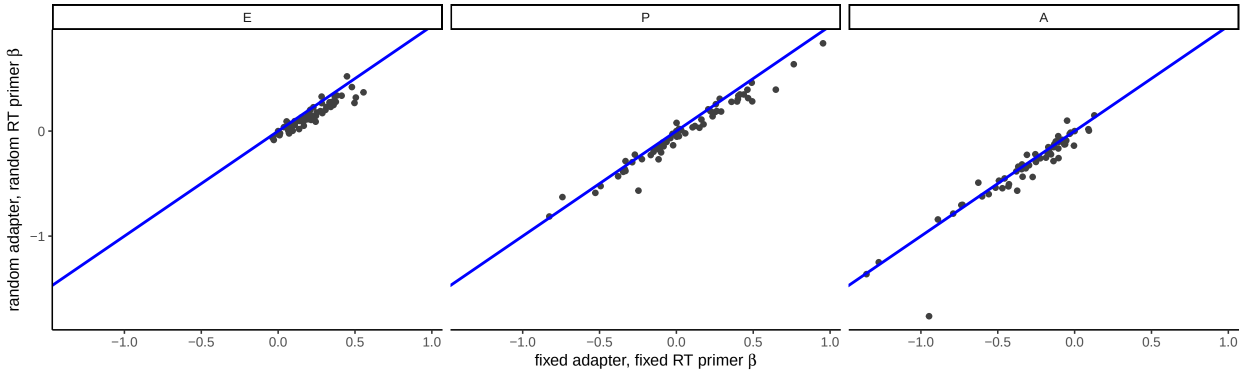

Supplementary Figure 4. Comparison of codon-level `choros` regression coefficients in an experimental validation dataset. Comparison of A-, P-, and E-site regression coefficients between two datasets generated from the same pool of ribosome footprints but using different library preparation protocols. A 1:1 line is shown in blue.

bioRxiv preprint doi: https://doi.org/10.1101/2023.02.21.529452; this version posted February 22, 2023. The copyright holder for this preprint (which was not certified by peer review) is the author/funder, who has granted bioRxiv a license to display the preprint in perpetuity. It is made available under aCC-BY 4.0 International license.

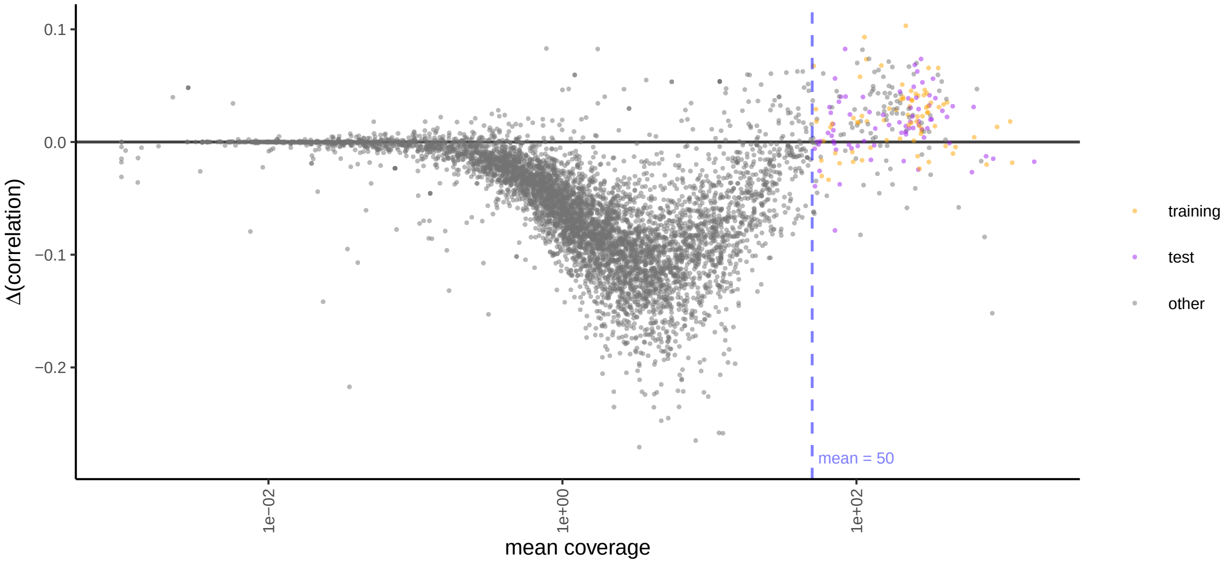

Supplementary Figure 5. Per-codon footprint counts on sparse transcripts may be untrustworthy. Correlation of footprint counts between library preparation protocols were computed on a per-transcript basis using raw and bias-corrected footprint counts. The y-axis reflects the difference in correlation between protocols when using bias-corrected footprint counts. Only transcripts with a mean coverage of >50 footprint counts per codon position tend to increase in similarity after bias correction.

bioRxiv preprint doi: https://doi.org/10.1101/2023.02.21.529452; this version posted February 22, 2023. The copyright holder for this preprint (which was not certified by peer review) is the author/funder, who has granted bioRxiv a license to display the preprint in perpetuity. It is made available under aCC-BY 4.0 International license.

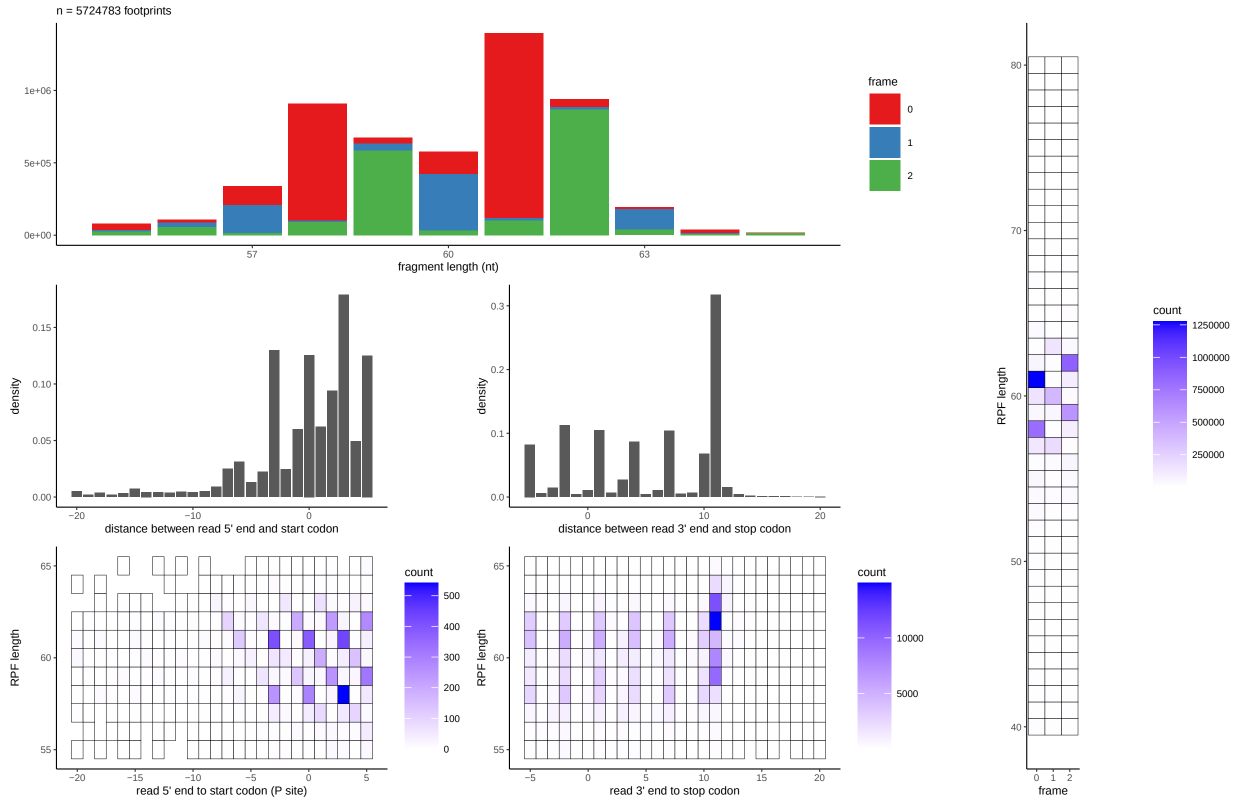

Supplementary Figure 6. Establishing leading and lagging A-site offset rules for disomes using start and stop codon metagene plots.
